# Supplementary material for: Mind the gap between recommendation and implementation—principles and lessons in the aftermath of incident investigations: a semi-quantitative and qualitative study of factors leading to the successful implementation of recommendations
Source: BMJ Open. 2014 May 29;4(5):e005326. doi: 10.1136/bmjopen-2014-005326 (PMC4039811; doi:10.1136/bmjopen-2014-005326)
Supplement: Author's manuscript [file bmjopen-2014-005326.draft_revisions.pdf]

BMJ Open

Mind the gap between recommendation and implementation - principles and learnings in the aftermath of incident investigations

Journal:	*BMJ Open*
Manuscript ID:	bmjopen-2014-005326
Article Type:	Research
Date Submitted by the Author:	23-Mar-2014
Complete List of Authors:	Wrigstad, Jonas; Department of Clinical Sciences Lund, Faculty of Medicine, Lund University, Department of Anesthesia and Intensive Care, Children's Hospital, Skåne University Hospital<br>Bergström, Johan; Lund University Centre for Risk Assessment and Management,<br>Gustafson, Pelle; Department of Clinical Sciences Lund, Faculty of Medicine, Lund University,
**Primary Subject Heading**:	Medical management
Secondary Subject Heading:	Qualitative research
Keywords:	QUALITATIVE RESEARCH, Change management < HEALTH SERVICES ADMINISTRATION & MANAGEMENT, Organisational development < HEALTH SERVICES ADMINISTRATION & MANAGEMENT

SCHOLARONE™
Manuscripts

For peer review only

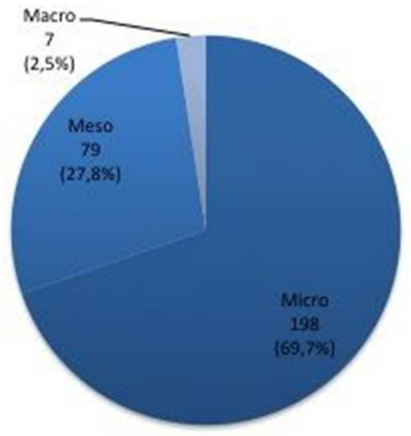

71x85mm (72 x 72 DPI)

For peer review only

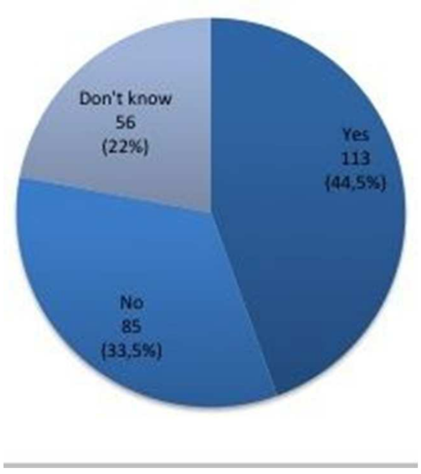

73x87mm (72 x 72 DPI)

For peer review only

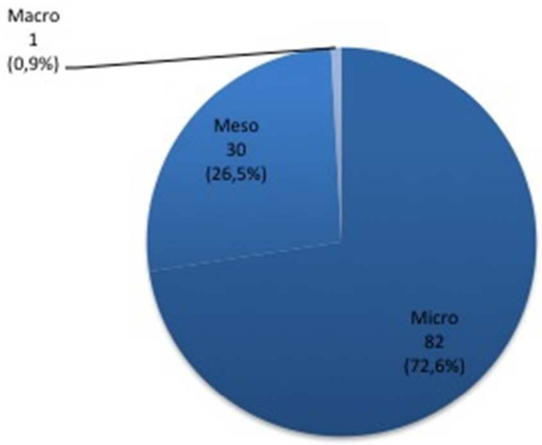

153x89mm (72 x 72 DPI)

For peer review only

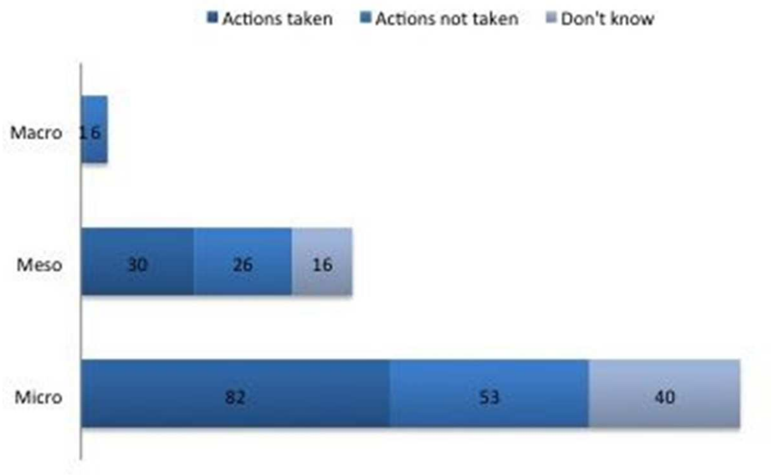

136x85mm (72 x 72 DPI)

For peer review only

TITLE PAGE

1. Title of the article:

Mind the gap between recommendation and implementation – principles and learning in the aftermath of incident investigations

2. Full name, postal adress, e-mail, telephone and fax numbers of the corresponding author:

Jonas Wrigstad
Department of Anesthesia and Intensive Care
Children's Hospital
Skåne University Hospital☐Lund
SE-22185 Lund
Sweden
E-mail: jonas.wrigstad@med.lu.se
Phone: +4646706200321
Fax: +4646178219

3. Full names, departments, institutions, city and country of all co-authors:

Johan Bergström, Lund University Centre for Risk Assessment and Management, Lund, Sweden

Pelle Gustafson, Department of Clinical Sciences, Faculty of Medicine, Lund University, Lund, Sweden

4. Keywords:

Management
Patient safety
Adverse events
Investigation

5. Word count:
4364

For peer review only

ABSTRACT

Background: Using incident investigations as a tool for improvement in patient safety management is both a well-established routine and a responsibility stated by law within the Swedish healthcare system. The main intention with the method in use is that the incident investigation promotes certain recommendations that through implementation in the organisation leads to improvement. This study seeks to identify the mechanisms behind successful implementation of such recommendations.

Methods: A method of three stages was used: a content analysis to code 284 recommendations in a sample of 55 incident investigations in a Swedish university hospital, semi-structured interviews with the commissioning bodies focusing on which recommendations that had been implemented and why, and finally a data analysis was conducted using the coded recommendations together with the interview data.

Results: A clear majority (69,7%) of the recommendations presented to the commissioning body were targeted towards the Micro-level of the organisation. In nearly half (44,5%) of all the recommendations actions had been taken and a clear majority (72,6%) of these were at the Micro-level. A change at the management position of the commissioning body meant that almost no further actions were taken. Separate actions were taken within the organisation that were not presented as recommendations in the incident investigations.

Conclusions: Two principles – ”close-in-space” and ”close-in-time” – seem to be important factors when closing the gap between recommendation and implementation. The Micro-level focus is expected because of the underlying method of analysis. Adverse events trigger organisational interaction regardless of the recommendations from incident investigations.

For peer review only

Strengths and limitations of this study

- **The results presented in this study shows the strength of using a design that combines content analysis with interviews to thereby get a deeper understanding of the different aspects of the data.**

- **The semi-structured nature of the interviews seemed to make the respondents willing to elaborate and reflect freely on both questions and follow-up questions which resulted in a substantial amount of qualitative data.**
- **The coding scheme in the Content analysis and the categories used in the Data analysis could possibly give a limited perspective of a more complex reality**

INTRODUCTION

When adverse events (AEs) occur in complex socio-technical healthcare systems, it's a challenge – if at all possible - to interpret the underlying causal factors. The importance of using past events to establish organisational learning is a non-controversial claim, but harder to institutionalise in practice.[1] Nevertheless, incident investigations have for decades been routine and seen upon as important tools in safety management, primarily to prevent similar events by promoting recommendations for ensuring continuous improvement.

Different organisations use different methods for their incident investigation activities and a majority, including healthcare, have adopted an underlying accident model in which recommendations made presupposes a stable causal structure of the system.[2, 3] Such a stable causal structure implies, as Lundberg et al point out,[2] that the recommendations are derived by identifying the root cause with no need for relating the specific recommendations to the damaged system as a whole. Johnson argues that the understanding of how certain recommendations are formulated is generally weak.[4] It has been shown that the time spent by the investigators on the activity of providing recommendations is surprisingly short, in comparison to other parts of the process.[5] Also, understanding the factors concerning implementation of recommendations from incident investigations is a process that so far has received limited focus in literature.[6, 7] The aim of this study therefore becomes to start filling the knowledge gap answering: what are the mechanisms behind successful implementation of the recommendation formulated in investigations of incidents in Swedish healthcare? The question follows Hollnagel's advice to search the positive aspects of safety rather than the negative ones.[8]

For peer review only

BACKGROUND

The Swedish healthcare systems regulatory authority at the time of the study, the National Board of Health and Welfare (NBoHaW), issues regulations controlling the responsibilities of the different healthcare givers in society, eg having an incident reporting system and performing incident investigations. Swedish law states that the responsibility for patient safety improvement within the organisation is placed with the separate healthcare givers.[9, 10] The law also states, that if an AE has resulted, or could have resulted, in a severe incident, this should be reported to the authority for a separate investigation. This investigation, the so called Lex Maria (LM)-investigation, is independent from the incident investigation by the healthcare provider. Deciding whether to report or not to the NBoHaW, is in Swedish healthcare almost exclusively a decision by the chief medical officer (CMO) in the organisation, although the CMO has neither formal legal authority nor responsibility as regards the safety level of the organisation.

A commissioning body (CB) initiates and frames the investigation, and is ultimately responsible for the follow-up of the recommendations from the incident investigation report. It could be argued that an investigation is not completed before a formal post-implementation follow-up has been done, but for clarification, in the present study we have left out the follow-up of the implemented recommendations, since the gap between recommendation and implementation is the focus of this study.

The analysis team, formed by the CB, will consist of at least one healthcare professional, trained in investigating AEs in the Swedish healthcare system. Since 2005, the methodological support for conducting the investigations is distributed by the Swedish Association of Local Authorities and Regions, and supported by the NBoHaW.[5]

In Swedish public healthcare, completed incident investigation reports are, after de-identification, publicly available. This is also the case for the LM-investigation made by the NBoHaW.

For peer review only

METHODS

In order to identify and analyse the success mechanisms behind positive implementation of recommendations following incident investigations in the Swedish healthcare system, a method of three stages was used. An initial content analysis was used to code the recommendations made in a sample of 55 incident investigations of adverse events in a Swedish university hospital. This was followed by semi-structured interviews of CBs focusing on which recommendations that had been implemented and why. Finally, a data analysis was conducted using the coded recommendations together with the interview data, which was used to isolate specific mechanisms contributing to successful implementation of recommendations. Due to the the semi-quantitative nature of the study, no attempt of a statistical analysis further than presented was done.

Content analysis

The first step was to sample a limited amount of completed incident investigations. In collaboration with the CMO at a Swedish university hospital, a frame was set to collect registered AEs, which led to incident investigations, through the incident reporting system of the hospital. The CMO was asked to judge from which year, following 2005 when the introduction of the manual for methodological support from the Swedish Association of Local Authorities and Regions was introduced, the hospital had a sufficient and qualified staff of incident investigators, working within the organisation and familiar with the methodology. Second, at least one year should have passed after completion of the incident investigation to give the hospital a reasonable chance for implementation of recommendations. Third, the selection of investigations should be linked to incidents in which the department of anaesthesia and intensive care at the hospital to some extent was involved. The reason behind this third selection was anaesthesiology being the profession of the main author ensuring (1) a comprehensive data set through his contacts with important actors, as well as (2) our understanding of the cases described through the subject matter expertise of the main author. This framing resulted in 55 separate incident investigations from January 2008 to December 2010. Twenty-three different CBs had initiated these 55 incident investigations. At this time, we had no knowledge about which CBs who had the same management position within the organisation, as they had when the incident investigation was initiated, or if there was a successor in that same position. We identified

For peer review only

the position from where the incident investigation was initiated, as this was the same position where the recommendations would be presented upon completion. Thus, continuity in management was of interest, not individuals.

Using the incident reporting system of the hospital, containing all registered AEs, the completed incident investigations were coupled to potentially existing additional investigations, e.g. LM-investigations.

All incident investigation reports and recommendations were numbered as they were received from the office of the CMO. Data from the reports was coded in a coding scheme focusing on the different CBs at the time of investigation, the ward from which the analysis was commissioned, time spent by the investigation team on conducting the investigation, the number of team members in the investigation team, the number of suggested recommendations, whether or not the findings of the investigation were reported by the hospital to the NBoHaW (as a LM-report).

Rasmussen and Svedung [11] shift the focus to not only include 'what' causal factors that get constructed, in the aftermath of adverse events, to also include 'where' in the organisational hierarchy that the constructed causal factors get located. We coded the reports regarding the hierarchical level being the target of recommendation according to a micro-meso-macro perspective.[12] This was done in order to find potential correlations between the hierarchical level and the likelihood of the recommendation being implemented. Micro was judged as a recommendation where it was possible for the CB to implement this solely within its own department without major constraints, e.g. local procedures, technical skills or staff issues. Meso was judged as a recommendation were the CB had to collaborate with a stakeholder outside of the own department, but within the boundaries of the hospital, e.g. another department or the hospital management. Macro was judged as a recommendation were the boundaries of the hospital had to be crossed, e.g. authorities, politicians, or pharmaceutical companies.

For peer review only

From the written reports it was not possible to gain knowledge to what extent the different recommendations had been implemented or not. This data was added to the coding scheme following the interviews.

Semi-structured interviews

The second part of this study consisted of an interview study with the different CBs at the hospital, to gain a deeper insight into which recommendations had been implemented and why. The interviews were semi-structured, as they focused on specific reports, but with the possibility for the respondents to reflect freely on the questions asked.

All of the CBs received written information prior to the interview about the background and aims of the project, and the main questions that formed the basis for the interview, in order to decide if they approved to participate. All the respondents were de-identified and given a random number. Twenty-two of 23 CBs (or their successors) approved through written consent to being interviewed. This made it possible to ask questions of interest in 50 of 55 incident investigation reports, with a total of 254 coded recommendations. Four of the 22 CBs delegated the interview to either the assistant director (2/4), or to the head advisor in patient safety (2/4). The interviews were all carried out during the period of April to September 2012. 20 of the interviews were audio recorded. In 2 of the interviews the respondents did not agree to audio recording and instead extensive notes were taken. All quotations presented here have been translated from Swedish to English by the author and the quotations are all tagged with the number of the coded respondent.

All interviews included a minimum of three questions (see below). Subsequent questions were asked depending on the answers given by the respondents.

1. Have you taken part of this incident investigation report with attention to the recommendations *before* this study?
2. Which recommendations from the incident report have been *implemented* in the organisation?
3. Have, to your knowledge, any *alternative* actions been taken within the organisation because of the incident investigation report that were not presented as recommendations?

For peer review only - http://bmjopen.bmj.com/site/about/guidelines.xhtml

Due to the coincidental fact, that during the time-period framed by the CMO, the hospital had a system were the CB in one part of the hospital almost exclusively was the CMO, whereas in the other part the CB was the clinical head of the department, the interviews had to be conducted somewhat differently. A CMO in a Swedish hospital cannot also be clinical head of a department at the same time, or vice versa. Therefore, in the specific investigation reports where the CB was the CMO, interviews were also done with clinical heads of departments involved in the specific investigation. The reason for this was to get a deeper knowledge of how far the implementation process had developed regarding recommendations to the different departments.

Data analysis

The analysis was conducted as a generalization using the interview data to seek naturalistically generalized factors explaining the results of the content analysis.[13, 14]

Before the naturalistic generalization commenced, the coding scheme was extended with the different answers regarding if action had been taken or not on specific recommendations. We used three categories in this study:

A – actions have been taken and initiated/completed regarding the recommendation

B – actions have not been taken regarding the recommendation

C – no knowledge if actions have been taken

Due to the fact that many clinical heads of department would be interviewed about the same specific incident investigation, a decision had to be made if answers would fall into categories that were contradictory. It then became important to follow up with asking the clinical head answering according to category A how and when the particular recommendation had resulted in actions.

In the search for generalized patterns we analysed the interview data asking a number of questions: Why did the distribution between micro, meso and macro look the way it did? What was the connection with successful implementation, and why? What aspects of successful implementation of recommendations were not captured in the content analysis,

For peer review only

and why? Did, or did not, factors such as the position of the CB or the time spent by the analysis team, influence the likelihood of the suggested recommendations being implemented?

FINDINGS

Content analysis of incident investigation reports

In this section, we present derived semi-quantitative data from the content analysis of the 55 incident investigations.

39 of the 55 AEs were subject to both an incident investigation by the hospital and to a LM-investigation by the authorities, suggesting that the degree of severity in a majority of the events had surpassed a limit stated by the authorities. Implementations of recommendations from the LM-investigations were not analysed in this study.

The CBs of the 55 incident investigations were similarly distributed between the CMOs (n=29) and the heads of departments (n=26).

The average number of team members per investigation was 2.7, and the duration of an investigation varied from 12 man-hours to 150 man-hours, where both of these findings are in the same range that have been shown by Rollenhagen for typical investigations in patient safety.[5]

Among the 55 incident investigations 289 separate recommendations were identified, where 5 recommendations were not coded because of our uncertainty of what the investigators actually meant in their written report. Thus 284 coded recommendations remained and their distribution in the organisational hierarchy is shown in figure 1. As mentioned previously, questions of interest were later raised during the interviews in 254 of these coded recommendations.

Figure 1. The distribution of the recommendations according to the three hierarchical levels

For peer review only

In the following sections both semi-quantitative and qualitative data, including the categories from the content analysis and quotations from the interviews of the CBs, will be presented in order to identify mechanisms important (or not) for the successful implementation of recommendations.

Management continuity

During the interviews, we found that the hospital, since commissioning the investigations, had appointed a different CMO for all of the 29 incident investigations, and different clinical heads of department, to the ones actually commissioning the investigations, in 41 cases of the broadened interview material, where one incident investigation could involve a number of interviews with directors of department. When the question "*have you taken part of this incident investigation report with attention to the recommendations before this study?*" was raised, the new CMO had taken part of 3/29 and the new clinical heads of department 6/41. As one of the CBs noted:

> "One could have a system where the CMO is a bit more meticulous and does a follow-up of the incident investigations to see what happened. It could be more of supervising position than it is today, but there is no time for that. That would probably be a part time job in itself or a substantially increased workload."(11.)

Overall, the respondents were concerned about the lack of knowledge regarding incident investigation reports completed before they came into their management position.

> ”I have not informed myself about past events, but that illustrates two important things, according to myself, that we use the results from the incident investigations too scantly and there is not enough follow-up. (…) But I think the most important matter is – these are historical cases and if one hasn't been clinically involved it's a problem with commitment – that there is a follow-up on the recommendations so that something does happen…" (2.)

Nowhere in the organisation, we found a good system of keeping record of which actions had been taken on the recommendations from the incident investigations. To a varying degree, the respondents had been able to find information on what kind of actions had been

For peer review only

taken. As shown in figure 2, after all interviews with the interpretation approach mentioned above, we found that for 44,5% of the recommendations actions had been taken, for 33,5% actions had not been taken and for 22,0% of the recommendations made our respondents were not able to tell us whether actions had been taken.

> ”No, note that there isn’t a single one of these incident investigations I’ve known about. (…) I’ve talked to my assistant director, dr (…), and to the member of staff responsible for the departments incident reporting, nurse (…), to collect some information” (22.)

Figure 2. Distribution of actions taken for all recommendations

The position of the CB

Whether the CB was a CMO or head of department did not seem to influence the process of implementation. When it was a CMO actions had been taken in 55/128 (42,9%) of completed investigations, and when it was a head of department it was 58/126 (46,0%).

> “…and when many departments are involved in the adverse event it doesn’t work with just one director of department being the commissioning body…But it’s also complicated to hand this over to the chief medical officer because it often has a tendency to come to nothing when many actors are involved. Who takes the responsibility?” (11.)

Micro, meso or macro

In the cases where actions have been taken (A), the interviews showed that a majority were on a Micro-level. In figure 3 the distribution between the three hierarchical levels are shown based on the total number of implemented recommendations (113) and in figure 4 the distribution is also shown in relation to the total number of recommendations made for each of the three hierarchical levels.

Figure 3. Actions taken as distributed on the three hierarchical levels

For peer review only

Figure 4. Actions taken or not for all recommendations as distributed on the hierarchical levels

> ”Yes, actions have been taken. We’ve written a new document about this procedure, that I have right in front of me, so that I can remember everything that has been done…and regarding *that* matter we’ve put it on the checklist and the surgeon must ask before surgery whether procedures have been followed.(…)This was a very easy and straightforward thing to solve, one could say. There was one thing that had gone wrong and we tried to fix it …and others weren’t involved”. (4.)

The event itself as a trigger for change

In 19 of 50 cases, the interviews showed that the AE had initiated organisational actions that were not presented as recommendations in the reports. It seemed that the incident investigations in these cases worked more as an incentive to change things, but on the initiative from the management, rather than from the analysis team.

> ”So you see, despite numerous meetings and brain storming back and forth I still believe that all of this was completely off target. (…) So in this case we did this formalistic play, which was good, but then we resigned a bit. Thereafter, among the senior colleagues, we drew a pragmatic conclusion and went on. There was someone who quoted Shakespeare at the time; ’Much ado about nothing’ or something like that…” (10.)

> “…then some of us decided, within the department, to start a minor recurring training course. (…) You see, it often comes down to quite strange results if we aren’t part of the changing process. (…) And when the colleagues ‘over there’ gained some knowledge about this matter things definitely got better, at least from my point of view. (…) Today this way of working is almost self-driven and I see it as a result completely independent of the investigation.” (16.)

The time spent by the investigation team

In 7 of 50 incident investigations it was not possible to find out the time spent by the investigating team on conducting the investigation. In 43 of 50 investigations, which consisted of 217 recommendations, there was a spread in duration from 12 man-hours to

For peer review only

150 man-hours. By arbitrarily grouping different investigations after time spent on the investigation, we wanted to examine if duration was a factor for implementation and at what level.

The groups consisted of short duration <40h, medium duration 41 to 80h and long duration >81h. We found that in the group with short duration, actions had been taken in 25/55 with 21/25 actions being on a Micro-level, in the group with medium duration actions had been taken in 43/116 with 28/43 actions on Micro-level, and in the long-duration group actions had been taken in 24/46 with 20/24 actions on Micro-level. Thus, with the methods in use in the hospital, the duration of an investigation differed with more than a factor 10, without time consumption seeming to influence the actions that were taken to meet the recommendations made, or at what level.

DISCUSSION

This study shows that a clear majority of the recommendations presented to the CB are targeted towards the Micro-level of the organisation, even when the investigating team spent a considerable amount of time. Our conclusion of why this is the case is not that the Micro-level is necessarily the most meaningful target of intervention, but rather that it reflects the investigating teams' understanding of how incidents happen. This is well summarised in Hollnagel's two principles: WYLFIWYF ("What You Look For Is What You Find") and WYFIWYF ("What You Find Is What You Fix") [2, 7, 15]. In this study what the investigators have been looking for, in terms of causal relations, is intimately linked to the linear causation constructed not as much by the investigators themselves as by the method available to them. A linear incident model, as the one inherent in the method provided by the Swedish Association of Local Authorities and Regions [5], used in the investigations studied, identifies certain problems as relevant targets of intervention. This is not the first study supporting the conclusion that linear incident investigation methods tend to locate causal explanations at the Micro-level of the organisational hierarchy [16], although we also see that what is found is not always fixed and it is not always the recommendations written in the reports that decide what will be fixed.

For peer review only

In the literature regarding healthcare system safety, much focus has been directed towards the sharp end, such as transition in care: the changing of shifts, change of ward and change of level in care.[17-19] Based on the findings from this study, we argue that in order to understand successful implementation of recommendations after analyses of AEs, important factors are also to be found at the blunt end of the organisation, such as changes in the positions in management or management continuity. Thereby saying that if the individual in a management position was a successor to the original commissioning body, there was almost no previous knowledge of an existing completed investigation, and understandably, hardly any further actions were taken than had already been done by the predecessor.

Consequently, two principles - “close-in-space” and “close-in-time” - seem to be important factors when closing the gap between recommendation and implementation using a model such as in this university hospital.

The finding that the event itself triggers organisational interventions regardless of the recommendations made in the incident investigation requires some further elaboration. Not only could this be interpreted as a finding that lowers the organisational mandate of the analysis process, but it also complicates the process of conducting the analysis, especially if using a model assuming a stable causal structure. If organisational interventions get initiated simply as a result of the event this means that the organisation essentially goes through a qualitative process of change as a result of the event. Consequently this implies that the organisation is qualitatively different after the event than it was before.

Since the organisation did not keep record of which recommendations had been taken action on, our findings rely almost entirely on the responses from the interviews. This in itself could give a hint of some uncertainty about the reliability of the analysis, but could on the other hand raise some concern about how incident investigations are used in the organisations work with patient safety improvement. Based on the interviews, nearly half of all the recommendations had been implemented, almost regardless of the organisation’s perceived severity of the adverse event. A clear majority of these recommendations were at the Micro-level with hardly any difference in actions taken depending on the management position of the CB.

For peer review only - http://bmjopen.bmj.com/site/about/guidelines.xhtml

The focus on success mechanisms also becomes a focus on system vulnerabilities and potential improvement. The finding that suggested actions are most likely to get implemented following a “close in time”- and “close in space”-principle can indeed guide future work to improve the system of learning from AEs in Swedish healthcare. We suggest that future research and projects aimed at improving the quality of the system focus on:

- Developing means to institutionalise an organisational memory of AEs and the analyses following them so that the system becomes less sensitive to management continuity.
- Pushing the target of analysis following AEs towards levelling the balance of suggested actions more between the Micro, Meso and Macro levels of the organisation. This requires a method for analysis which directs the focus of analysis to interactions and relations at higher organisational levels, but also investigation teams with basic competence in safety science and interpretation of complex systems.
- The gap between investigation team and investigated organisation. Based on the finding that actions beyond those suggested in the formal analyses are taken following AEs we suggest future work to be done studying how the dialogue between analysis team and the organisation being the target of the analysis can be enhanced.
- Learning from incidents beyond the formalised system. We suggest future research to be conducted with a focus on possible story telling of past incidents in healthcare organisations. Is it actually so that there are many lessons learned that never get brought up in formal investigations, but that nevertheless becomes a part of the organisational memory and everyday behaviour?

CONCLUSIONS

This study seeks to interpret factors that lead to successful implementation of recommendations suggested in incident investigations following AEs in a Swedish university hospital. Based on the findings the following conclusions are drawn:

- Continuity in management is an important factor for successful implementation of recommendations = Close-in-time.

For peer review only

- A clear majority (69,7 %) of the recommendations presented in the studied incident investigations are targeted towards a Micro-level of the organisation. The same applies to the recommendations that actually get implemented: 72,6 % in this study = Close-in-space.
- The Micro-level focus of the investigations is expected given the linear causal model underlying the method of analysis. For recommendations to be targeted towards Meso- and Macro-levels of the organisation the model used for investigation needs to seek explanatory factors at the level of organisational interactions and relations.
- The AE itself triggers organisational interventions regardless of the recommendations made in the incident investigations.
- The time spent by the investigation team or the position of the CB were factors which did not stand out as contributing to successful implementation of recommendations.

FOOTNOTES

Contributors: JW developed the study, collected, analysed and interpreted the data and wrote the manuscript. JB and PG contributed in the study design, analysis of the data and critical revision of the manuscript for important intellectual content.

Acknowledgments: The authors would like to thank the staff at the office of the CMO for their contribution by providing all the necessary incident investigation reports.

Competing interests: None.

Ethics approval: The study was approved by The Regional Ethical Review Board in Lund, Sweden.

Funding: The project was partially funded by a grant, but with no further involvement, from The Swedish National Patient Insurance.

Data sharing: No additional data available.

REFERENCES

1. Woods DD, Cook RI. Nine steps to move forward from error. *Cogn Technol Work* 2002;**4**(2):137-44.

For peer review only

2. Lundberg J, Rollenhagen C, Hollnagel E. What-You-Look-For-Is-What-You-Find--The consequences of underlying accident models in eight accident investigation manuals. *Saf Sci* 2009;**47**(10):1297-311.

3. Rasmussen J, Nixon P, Warner F. Human error and the problem of causality in analysis of accidents [and discussion]. *Philos Trans R Soc London B Biol Sci* 1990;**327**(1241):449-62.

4. Johnson CW. Failure in safety-critical systems: a handbook of accident and incident reporting. University of Glasgow Press, Glasgow, Scotland, 2003:541-623

5. Rollenhagen C, Westerlund J, Lundberg J et al. The context and habits of accident investigation practices: A study of 108 swedish investigators. *Saf Sci* 2010;**48**(7):859-67.

6. Cedergren A. Implementing recommendations from accident investigations: A case study of inter-organisational challenges. *Accid Anal Prev* 2013, Apr;**53**:133-41.

7. Lundberg J, Rollenhagen C, Hollnagel E. What you find is not always what you fix—how other aspects than causes of accidents decide recommendations for remedial actions. *Accid Anal Prev* 2010;**42**(6):2132-9.

8. Hollnagel E. Resilience engineering in practice: A guidebook. Ashgate Publishing, Ltd.; 2011:xxix-xxxix

9. The Swedish Patient Safety Act (SFS 2010:659). http://www.riksdagen.se/sv/Dokument-Lagar/Lagar/Svenskforfattningssamling/Patientsakerhetslag-2010659_sfs-2010-659/ (accessed 4 Nov 2013)

10. The National Board of Health and Welfares management system for systematic quality work (SOSFS 2011:9). http://www.socialstyrelsen.se/sosfs/2011-9/allaversioner2011-9 (accessed 4 Nov 2013)

11. Rasmussen J, Svedung I. Proactive risk management in a dynamic society. Karlstad: Swedish Rescue Services Agency 2000.

12. Cedergren A, Petersen K. Prerequisites for learning from accident investigations--a cross-country comparison of national accident investigation boards. *Saf Sci* 2011;**49**(8):1238-45.

13. Cresswell JW. Qualitative inquiry and research design: Choosing among five approaches. Lincoln: Sage Publications 2007.

14. Stake RE. The art of case study research. Thousand Oaks: Sage Publications 1995.

For peer review only

15. Hollnagel E. Investigation as an impediment to learning. In: Hollnagel E, Nemeth CP, Dekker SWA, eds. Resilience Engineering Perspectives: Remaining Sensitive to the Possibility of Failure, vol. 1. Aldershot: Ashgate Publishing Limited 2008: 259-268.

16. Dekker SWA, Cilliers P, Hofmeyr JH. The complexity of failure: Implications of complexity theory for safety investigations. Safety Science 2011.

17. Cook RI, Render M, Woods DD. Gaps in the continuity of care and progress on patient safety. *BMJ* 2000;**320**(7237):791-4.

18. Flin RH, O'Connor P, Crichton M. Safety at the sharp end: Training non-technical skills. Ashgate Publishing; 2008.

19. Perry SJ, Wears RL, Patterson ES. High-hanging fruit: Improving transitions in health care. Advances in Patient Safety: New Directions and Alternative Approaches 2008;**3**.

For peer review only

BMJ Open

Mind the gap between recommendation and implementation – principles and learnings in the aftermath of incident investigations. A semi-quantitative and qualitative study in search for factors that lead to successful implementation of recommendations.

Journal:	*BMJ Open*
Manuscript ID:	bmjopen-2014-005326.R1
Article Type:	Research
Date Submitted by the Author:	02-May-2014

Complete List of Authors:	Wrigstad, Jonas; Lund University, Department of Clinical Sciences, Lund, Sweden<br>Bergström, Johan; Lund University, Centre for Risk Assessment and Management, Lund, Sweden<br>Gustafson, Pelle; Lund University, Department of Clinical Sciences, Lund, Sweden

**Primary Subject Heading**:	Medical management
Secondary Subject Heading:	Qualitative research
Keywords:	QUALITATIVE RESEARCH, Change management < HEALTH SERVICES ADMINISTRATION & MANAGEMENT, Organisational development < HEALTH SERVICES ADMINISTRATION & MANAGEMENT

SCHOLARONE™ Manuscripts

For peer review only

TITLE PAGE

1. Title of the article:

Mind the gap between recommendation and implementation – principles and learnings in the aftermath of incident investigations. A semi-quantitative and qualitative study in search for factors that lead to successful implementation of recommendations.

2. Full name, postal address, e-mail, telephone and fax numbers of the corresponding author:

Jonas Wrigstad
Department of Anesthesia and Intensive Care
Children's Hospital
Skåne University Hospital Lund
SE-22185 Lund
Sweden
E-mail: jonas.wrigstad@med.lu.se
Phone: +4646706200321
Fax: +4646178219

3. Full names, departments, institutions, city and country of all co-authors:

Johan Bergström, Lund University, Centre for Risk Assessment and Management, Lund, Sweden

Pelle Gustafson, Lund University, Department of Clinical Sciences, Lund, Sweden

4. Keywords:

Management
Patient safety
Adverse events
Investigation

For peer review only

ABSTRACT

Objectives: Using incident investigations as a tool for improvement in patient safety management is both a well-established routine, and a responsibility stated by Swedish law. The main intention with the method is to promote certain recommendations that through implementation in the organisation lead to improvement. This study seeks to identify the mechanisms behind successful implementation of such recommendations.

Setting: A university hospital in southern Sweden

Participants: From the hospitals incident reporting system a sample of 55 incident investigations from 2008 to 2010 were selected by staff at the chief medical officer of the organisation. These investigations were initiated by 23 different commissioning bodies and contained 289 separate recommendations. We used a three-stage method: first a content analysis to code the recommendations, then semi-structured interviews with the commissioning bodies focusing on which recommendations that had been implemented and why, and third, a data analysis of the coded recommendations together with data from the interviews.

Results: We found that a clear majority (70 %) of the recommendations presented to the commissioning bodies were targeted towards the micro-level of the organisation. In nearly half (45 %) of all the recommendations, actions had been taken and a clear majority (73 %) of these were at the micro-level. A change at the management position of the commissioning body meant that almost no further actions were taken. Separate actions, free-standing from and independent of the incident investigations, were often taken within the organisation.

Conclusions: We conclude that two principles (”close-in-space” and ”close-in-time”) seem to be important factors when bridging the gap between recommendation and implementation. The micro-level focus is expected because of the method of investigation used. Adverse events trigger organisational interaction independently of incident investigations.

For peer review only

Strengths and limitations of this study

- The results presented in this study shows the strength of using a design that combines content analysis with interviews to thereby get a deeper understanding of the different aspects of the data.
- The semi-structured nature of the interviews seemed to make the respondents willing to elaborate and reflect freely on both questions and follow-up questions which resulted in a substantial amount of qualitative data.
- The coding scheme in the Content analysis and the categories used in the Data analysis could possibly give a limited perspective of a more complex reality

INTRODUCTION

When adverse events (AEs) occur in complex socio-technical healthcare systems, it is a challenge – if at all possible - to interpret the underlying causal factors. The importance of using past events to establish organisational learning is a non-controversial claim, but harder to institutionalise in practice.[1] Nevertheless, incident investigations have for decades been routine and seen upon as important tools in safety management, primarily to prevent similar events by promoting recommendations for ensuring continuous improvement.

Different organisations use different methods for their incident investigation activities and a majority, including healthcare, have adopted an underlying accident model in which recommendations made presupposes a stable causal structure of the system.[2, 3] Such a stable causal structure implies that the recommendations are derived by identifying the root cause with no need for relating the specific recommendations to the damaged system as a whole.[2] Johnson argues that the understanding of how certain recommendations are formulated is generally weak.[4] It has been shown that the time spent by the investigators on the activity of providing recommendations is surprisingly short, in comparison to other parts of the process.[5] Furthermore, which factors that govern a successful implementation of recommendations has so far received limited focus in literature.[6, 7]

For peer review only

The aim of this study was to start filling this knowledge gap by analysing the mechanisms behind successful implementation of recommendations formulated in investigations of incidents in Swedish healthcare. The approach follows Hollnagel's advice to search the positive aspects of safety rather than the negative ones.[8]

BACKGROUND

The Swedish healthcare systems regulatory authority at the time of the study, the National Board of Health and Welfare (NBoHaW), has issued regulations controlling the responsibilities of the different healthcare givers, e.g. using an incident reporting system and performing incident investigations. Swedish law states that the responsibility for patient safety improvement is placed within the organisation itself of the separate healthcare givers.[9, 10] The law also states that if an AE has resulted, or could have resulted, in a severe incident, this should be reported to the regulatory authority for a separate investigation. This investigation, the so called Lex Maria (LM)-investigation, is independent from the incident investigation by the healthcare provider. Deciding whether to report or not to the NBoHaW, is almost exclusively a decision by the chief medical officer (CMO) of the organisation, although the CMO has neither formal legal authority nor responsibility as regards the safety level of the organisation.

A commissioning body (CB) initiates and frames the incident investigation, and is ultimately responsible for the follow-up of the recommendations from the report.
The analysis team, formed by the CB, will consist of at least one healthcare professional, trained in investigating AEs in the Swedish healthcare system. Since 2005, the methodological support for conducting the investigations is distributed by the Swedish Association of Local Authorities and Regions (SALAR), and supported by the NBoHaW.[5] In Swedish healthcare, completed incident investigation reports are, after de-identification, publicly available, as are LM-investigations made by the NBoHaW.

METHODS

We used a three-stage method. First, we did a content analysis to code the recommendations in a sample of 55 incident investigations of AEs in a Swedish university

For peer review only

hospital. We then did semi-structured interviews of CBs focusing on which recommendations that had been implemented and why. Finally, we conducted a data analysis using the coded recommendations together with the interview data, which was used to isolate specific mechanisms contributing to successful implementation of recommendations. Due to the semi-quantitative nature of the study, we did no statistical analyses further than presented.

Content analysis

The first step was to sample a limited amount of completed incident investigations. In collaboration with the CMO at a Swedish university hospital, a frame was set to collect registered AEs, which led to incident investigations, through the incident reporting system of the hospital. The CMO was asked to judge from which year, following 2005 when the introduction of the manual for methodological support from the SALAR was introduced, the hospital had a sufficient and qualified staff of incident investigators who were working within the organisation and familiar with the methodology. Second, at least one year should have passed after completion of the incident investigation to allow a reasonable chance for implementation of recommendations. Third, the selection of investigations should be linked to incidents in which the department of anaesthesia and intensive care at the hospital to some extent was involved. The reason behind this third selection was the main author being an anaesthesiologist, ensuring 1) a comprehensive data set through contacts with important actors, as well as 2) being able to fully understand the cases and investigations, regardless of complexity. This framing resulted in 55 separate incident investigations from January 2008 to December 2010, initiated by 23 different CBs. We also identified the position from where the incident investigation was initiated, as this was the same position where the recommendations would be presented upon completion. Thus, continuity in management was of interest, not individuals.

Using the incident reporting system of the hospital, containing all registered AEs, the completed incident investigations were coupled to potentially existing additional investigations, e.g. LM-investigations.

For peer review only

All incident investigation reports and recommendations were numbered as they were received from the office of the CMO. Data from the reports was coded in a scheme focusing on the different CBs at the time of investigation, the ward from which the analysis was commissioned, time spent by the investigation team on conducting the investigation, the number of team members, the number of suggested recommendations, and whether or not the findings of the investigation were reported by the hospital to the NBoHaW (as a LM-investigation).

Rasmussen and Svedung [11] shift the focus to not only include 'what' causal factors that get constructed in the aftermath of adverse events, to also include 'where' in the organisational hierarchy that the constructed causal factors get located. We therefore coded the reports regarding the hierarchical level being the target of recommendation according to a micro-meso-macro perspective.[12] This was done in order to find potential correlations between the hierarchical level and the likelihood of the recommendation being implemented. Micro was judged as a recommendation where it was possible for the CB to implement this entirely within its own department without major constraints, e.g. local procedures, technical skills or staff issues. Meso was judged as a recommendation were the CB had to collaborate with a stakeholder outside of the own department, but within the boundaries of the hospital, e.g. another department or the hospital management. Macro was judged as a recommendation were the boundaries of the hospital had to be crossed, e.g. authorities, politicians, or pharmaceutical companies.

From the written reports it was not possible to gain knowledge to what extent the different recommendations had been implemented or not. This data was added to the coding scheme following the interviews.

Semi-structured interviews

The second part of this study consisted of an interview study with the different CBs at the hospital, to gain a deeper insight into which recommendations had been implemented and why. The interviews were semi-structured, as they focused on specific reports, but with the possibility for the respondents to reflect freely on the questions asked.

For peer review only

All of the CBs received written information prior to the interview about the background and aims of the project, as well as the main questions that formed the basis for the interview. All respondents were de-identified and given a random number. Twenty-two of 23 CBs (or their successors) approved through written consent to being interviewed. This made it possible to ask questions of interest in 50 of 55 incident investigation reports, with a total of 254 coded recommendations. Four of the 22 CBs delegated the interview to either the assistant director (2/4), or to the head advisor in patient safety (2/4). The interviews were all carried out during the period of April to September 2012 by the first author (JW) at a place suggested by the respondent. Twenty of the interviews were audio recorded. In 2 of the interviews the respondents did not agree to audio recording and instead extensive notes were taken. All quotations presented here have been translated from Swedish to English by the first author and the quotations are all tagged with the number of the coded respondent.

All interviews included a minimum of three questions (see below). Subsequent questions were asked depending on the answers given by the respondents.

1. Have you taken part of this incident investigation report with attention to the recommendations *before* this study?
2. Which recommendations from the incident report have been *implemented* in the organisation?
3. Have, to your knowledge, any *alternative* actions been taken within the organisation because of the incident investigation report that were not presented as recommendations?

Due to the coincidental fact, that during the time-period framed by the CMO, the hospital had a system were in one part of the hospital, the CB almost exclusively was the CMO, whereas in the other part the CB was the clinical head of the department, the interviews had to be conducted somewhat differently. A CMO in a Swedish hospital cannot also be clinical head of a department at the same time, or vice versa. Therefore, in the investigation reports where the CB was the CMO, interviews were also done with clinical heads of departments involved. The reason for this was to get a deeper knowledge of how far the implementation process had developed regarding recommendations to the different departments.

For peer review only

Data analysis

The analysis was conducted as a generalization using the interview data to seek naturalistically generalized factors explaining the results of the content analysis.[13, 14]

Before the naturalistic generalization commenced, the coding scheme was extended with the different answers regarding if action had been taken or not on specific recommendations. We used three categories in this study:

A – actions have been taken and initiated/completed regarding the recommendation

B – actions have not been taken regarding the recommendation

C – no knowledge if actions have been taken

Due to the fact that many clinical heads of department would be interviewed about the same specific incident investigation, a decision had to be made if answers would fall into categories that were contradictory. It then became important to follow up with asking the clinical head answering according to category A how and when the particular recommendation had resulted in actions.

We analysed interview data in particular search for generalized patterns: Why did the distribution between micro, meso and macro look the way it did? What was the connection with successful implementation, and why? What aspects of successful implementation of recommendations were not captured in the content analysis, and why? Did, or did not, factors such as the position of the CB or the time spent by the analysis team, influence the likelihood of the suggested recommendations being implemented?

RESULTS

Content analysis of incident investigation reports

39 of the 55 AEs were subject to both an incident investigation by the hospital and to a LM-investigation by the authorities, suggesting that the degree of severity in a majority of the events had surpassed a limit stated by the authorities. Implementations of recommendations from the LM-investigations were not analysed in this study.

For peer review only

The CBs of the 55 incident investigations were similarly distributed between the CMOs (n=29) and the heads of departments (n=26).

The average number of team members per investigation was 2.7, and the duration of an investigation varied from 12 man-hours to 150 man-hours, where both of these findings are in the same range that have been shown by Rollenhagen for typical investigations in patient safety.[5]

Among the 55 incident investigations 289 separate recommendations were identified, where 5 recommendations were not coded because of our uncertainty of what the investigators actually meant in their written report. Thus 284 coded recommendations remained and, as mentioned previously, questions of interest were raised during the interviews in 254 of these coded recommendations where their distribution in the organisational hierarchy is shown in table 1.

Table 1. The distribution of the recommendations according to the three hierarchical levels as well as whether they have been reported as having resulted in actions.

Hierarchical level	No of recommendations for which actions have been taken	No of recommendations for which actions have not been taken	No of recommendations for which there is no knowledge if actions have been taken	Total number
**Macro**	1	6	0	**7**
**Meso**	30	26	16	**72**
**Micro**	82	53	40	**175**
**Total number**	**113**	**85**	**56**	

In the following sections both semi-quantitative and qualitative data, including the categories from the content analysis and quotations from the interviews of the CBs, will be presented in order to identify mechanisms important (or not) for the successful implementation of recommendations.

For peer review only

Management continuity

From the interviews, we found that the hospital, since commissioning the investigations, had appointed a new CMO for all of the 29 incident investigations, and new clinical heads of department in 41 cases of the broadened interview material, where one incident investigation could involve a number of interviews with directors of department. When the question *"have you taken part of this incident investigation report with attention to the recommendations before this study?"* was raised, the new CMO had taken part of 3/29 and the new clinical heads of department 6/41. As one of the CBs noted:

> "One could have a system where the CMO is a bit more meticulous and does a follow-up of the incident investigations to see what happened. It could be more of supervising position than it is today, but there is no time for that. That would probably be a part time job in itself or a substantially increased workload."(11.)

Overall, the respondents were concerned about the lack of knowledge regarding incident investigation reports completed before taking on their management position.

> "I have not informed myself about past events, but that illustrates two important things, according to myself, that we use the results from the incident investigations too scantly and there is not enough follow-up. (…) But I think the most important matter is – these are historical cases and if one hasn't been clinically involved it's a problem with commitment – that there is a follow-up on the recommendations so that something does happen…" (2.)

Nowhere in the organisation, we found a good system of keeping record of which actions had been taken on the recommendations from the incident investigations. To a varying degree, the respondents had been able to find information on what kind of actions had been taken. As shown in table one, after all interviews with the interpretation approach mentioned above, we found that for 45 % of the recommendations actions had been taken, for 33 % actions had not been taken, and for 22 % of the recommendations made our respondents were not able to tell us whether actions had been taken.

For peer review only

”No, note that there isn’t a single one of these incident investigations I’ve known about. (…) I’ve talked to my assistant director, dr (…), and to the member of staff responsible for the departments incident reporting, nurse (…), to collect some information” (22.)

The position of the CB

Whether the CB was a CMO or head of department did not seem to influence the process of implementation. When it was a CMO actions had been taken in 55/128 (43 %) of completed investigations, and when it was a head of department it was 58/126 (46 %).

“…and when many departments are involved in the adverse event it doesn’t work with just one director of department being the commissioning body…But it’s also complicated to hand this over to the chief medical officer because it often has a tendency to come to nothing when many actors are involved. Who takes the responsibility?” (11.)

Micro, meso or macro

As seen in table one, in the cases where actions have been taken (A), the interviews showed that a majority were on a micro-level.

”Yes, actions have been taken. We’ve written a new document about this procedure, that I have right in front of me, so that I can remember everything that has been done…and regarding *that* matter we’ve put it on the checklist and the surgeon must ask before surgery whether procedures have been followed.(…) This was a very easy and straightforward thing to solve, one could say. There was one thing that had gone wrong and we tried to fix it …and others weren’t involved”. (4.)

The event itself as a trigger for change

In 19 of 50 cases, the interviews showed that the AE had initiated organisational actions that were not presented as recommendations in the reports. It seemed that the incident investigations in these cases worked more as an incentive to change things, but on the initiative from the management, rather than from the analysis team.

”So you see, despite numerous meetings and brain storming back and forth I still believe that all of this was completely off target. (…) So in this case we did this formalistic play,

For peer review only

which was good, but then we resigned a bit. Thereafter, among the senior colleagues, we drew a pragmatic conclusion and went on. There was someone who quoted Shakespeare at the time; 'Much ado about nothing' or something like that…" (10.)

"…then some of us decided, within the department, to start a minor recurring training course. (…) You see, it often comes down to quite strange results if we aren't part of the changing process. (…) And when the colleagues 'over there' gained some knowledge about this matter, things definitely got better, at least from my point of view. (…) Today this way of working is almost self-driven and I see it as a result completely independent of the investigation." (16.)

The time spent by the investigation team

In 7 of 50 incident investigations it was not possible to find out the time spent by the investigating team on conducting the investigation. In 43 of 50 investigations, which consisted of 217 recommendations, there was a spread in duration from 12 man-hours to 150 man-hours. By arbitrarily grouping different investigations after time spent on the investigation, we wanted to examine if duration was a factor for implementation and at what level.

The groups consisted of short duration <40h, medium duration 41 to 80h and long duration >81h. We found that in the group with short duration, actions had been taken in 25/55 with 21/25 actions being on a Micro-level, in the group with medium duration actions had been taken in 43/116 with 28/43 actions on Micro-level, and in the long-duration group actions had been taken in 24/46 with 20/24 actions on Micro-level. Thus, with the methods in use in the hospital, the duration of an investigation differed with more than a factor 10, without time consumption seeming to influence the actions that were taken to meet the recommendations made, or at what level.

DISCUSSION

This study has several strengths and limitations. The results presented shows the strength of using a design that combines content analysis with interviews to thereby get a deeper understanding of the different aspects of the data. The semi-structured nature of the

For peer review only

interviews seemed to make the respondents willing to elaborate and reflect freely on both questions and follow-up questions which resulted in a substantial amount of qualitative data. The coding scheme in the content analysis and the categories used in the data analysis could possibly give a limited perspective of a more complex reality. It could be argued that an investigation is not completed before a formal post-implementation follow-up has been done. However, we have not studied the effect of the implemented recommendations, since the gap between recommendation and implementation was the focus.

We dare not have an opinion as regards the possibility to draw general conclusions from this study. However, we have no reason to expect that our findings are unique to the speciality studied (anesthesiology), or the type of hospital studied (university hospital), and thus believe that our findings could be valid for other Swedish hospitals, and possibly also hospitals in countries with a similar system for investigations of AEs.

This study shows that a clear majority of the recommendations presented to the CB are targeted towards the micro-level of the organisation, even when the investigating team spent a considerable amount of time. Our conclusion of why this is the case is not that the micro-level is necessarily the most meaningful target of intervention, but rather that it reflects the investigating teams' understanding of how incidents happen. This is well summarised in Hollnagel's two principles: WYLFIWYF ("What You Look For Is What You Find") and WYFIWYF ("What You Find Is What You Fix").[2, 7, 15] In this study what the investigators have been looking for, in terms of causal relations, is intimately linked to the linear causation constructed not as much by the investigators themselves as by the method available to them. A linear incident model, as the one inherent in the method provided by the SALAR [5], used in the investigations studied, identifies certain problems as relevant targets of intervention. This is not the first study supporting the conclusion that linear incident investigation methods tend to locate causal explanations at the micro-level of the organisational hierarchy [16] although we also see that what is found is not always fixed and it is not always the recommendations written in the reports that decide what will be fixed.

In the literature regarding healthcare system safety, much focus has been directed towards the sharp end, such as transition in care: the changing of shifts, change of ward and change

For peer review only

of level in care.[17-19] Based on the findings from this study, we argue that in order to understand successful implementation of recommendations after analyses of AEs, important factors are also to be found at the blunt end of the organisation, such as changes in the positions in management or management continuity. Thereby saying that if the individual in a management position was a successor to the original commissioning body, there was almost no previous knowledge of an existing completed investigation, and understandably, hardly any further actions were taken than had already been done by the predecessor.

Consequently, two principles - “close-in-space” and “close-in-time” - seem to be important factors when closing the gap between recommendation and implementation using a model such as in this university hospital.

The finding that the event itself triggers organisational interventions regardless of the recommendations made in the incident investigation requires some further elaboration. Not only could this be interpreted as a finding that lowers the organisational mandate of the analysis process, but it also complicates the process of conducting the analysis, especially if using a model assuming a stable causal structure. If organisational interventions get initiated simply as a result of the event, this means that the organisation essentially goes through a qualitative process of change as a result of the event. Consequently this implies that the organisation is qualitatively different after the event than it was before.

Since the organisation did not keep record of which recommendations had been taken action on, our findings rely almost entirely on the responses from the interviews. This in itself could give a hint of some uncertainty about the reliability of the analysis, but could on the other hand raise some concern about how incident investigations are used in the organisations work with patient safety improvement. Based on the interviews, nearly half of all the recommendations had been implemented, almost regardless of the organisation’s perceived severity of the adverse event. A clear majority of these recommendations were at the micro-level with hardly any difference in actions taken depending on the management position of the CB.

For peer review only

The focus on success mechanisms also becomes a focus on system vulnerabilities and potential improvement. The finding that suggested actions are most likely to get implemented following a “close in time” and “close in space”-principle can indeed guide future work to improve the system of learning from AEs in Swedish healthcare. We suggest that future research and projects, aimed at improving the quality of the system, focus on 1) developing means to institutionalise an organisational memory of AEs and the analyses following them so that the system becomes less sensitive to management continuity, 2) pushing the target of analysis following AEs towards levelling the balance of suggested actions more between the micro, meso and macro levels of the organisation. This requires a method for analysis which directs the focus of analysis to interactions and relations at higher organisational levels, but also investigation teams with basic competence in safety science and interpretation of complex systems. Furthermore, 3) the gap between investigation team and investigated organisation. Based on the finding that actions beyond those suggested in the formal analyses are taken following AEs, we suggest future work to be done studying how the dialogue between analysis team and the organisation being the target of the analysis can be enhanced, and 4) learning from incidents beyond the formalised system. We suggest future research to be conducted with a focus on possible storytelling of past incidents in healthcare organisations. Is it actually so that there are many lessons learned that never get brought up in formal investigations, but that nevertheless becomes a part of the organisational memory and everyday behaviour?

CONCLUSIONS

This study seeks to interpret factors that lead to successful implementation of recommendations suggested in incident investigations following AEs in a Swedish university hospital. Based on the findings, we conclude that continuity in management is an important factor for successful implementation of recommendations (= close-in-time), as is that a clear majority of the recommendations presented in the studied incident investigations are targeted towards a micro-level of the organisation where the same applies to the recommendations that actually get implemented (= close-in-space). The micro-level focus of the investigations is expected given the linear causal model underlying the method of analysis. For recommendations to be targeted towards meso- and macro-levels of the organisation the model used for investigation needs to seek explanatory factors at the level

For peer review only

of organisational interactions and relations. Furthermore, the AE itself triggers organisational interventions regardless of the recommendations made in the incident investigations, and the time spent by the investigation team or the position of the CB were both factors which did not stand out as contributing to successful implementation of recommendations.

FOOTNOTES

Acknowledgments: The authors would like to thank the staff at the office of the CMO for their contribution by providing all the necessary incident investigation reports.

Funding: The project was partially funded by a grant, but with no further involvement, from The Swedish National Patient Insurance.

Contributors: JW developed the study, collected, analysed and interpreted the data and wrote the manuscript. JB and PG contributed in the study design, analysis of the data and revision of the manuscript.

Competing interests: None.

Ethics approval: The study was approved by The Regional Ethical Review Board in Lund, Sweden.

Data sharing: No additional data available.

For peer review only

REFERENCES

1. Woods DD, Cook RI. Nine steps to move forward from error. *Cogn Technol Work* 2002;**4**(2):137-44.

2. Lundberg J, Rollenhagen C, Hollnagel E. What-You-Look-For-Is-What-You-Find--The consequences of underlying accident models in eight accident investigation manuals. *Saf Sci* 2009;**47**(10):1297-311.

3. Rasmussen J, Nixon P, Warner F. Human error and the problem of causality in analysis of accidents [and discussion]. *Philos Trans R Soc London B Biol Sci* 1990;**327**(1241):449-62.

4. Johnson CW. Failure in safety-critical systems: a handbook of accident and incident reporting. University of Glasgow Press, Glasgow, Scotland, 2003:541-623

5. Rollenhagen C, Westerlund J, Lundberg J et al. The context and habits of accident investigation practices: A study of 108 swedish investigators. *Saf Sci* 2010;**48**(7):859-67.

6. Cedergren A. Implementing recommendations from accident investigations: A case study of inter-organisational challenges. *Accid Anal Prev* 2013, Apr;**53**:133-41.

7. Lundberg J, Rollenhagen C, Hollnagel E. What you find is not always what you fix—how other aspects than causes of accidents decide recommendations for remedial actions. *Accid Anal Prev* 2010;**42**(6):2132-9.

8. Hollnagel E. Resilience engineering in practice: A guidebook. Ashgate Publishing, Ltd.; 2011:xxix-xxxix

9. The Swedish Patient Safety Act (SFS 2010:659). http://www.riksdagen.se/sv/Dokument-Lagar/Lagar/Svenskforfattningssamling/Patientsakerhetslag-2010659_sfs-2010-659/ (accessed 4 Nov 2013)

10. The National Board of Health and Welfares management system for systematic quality work (SOSFS 2011:9). http://www.socialstyrelsen.se/sosfs/2011-9/allaversioner2011-9 (accessed 4 Nov 2013)

11. Rasmussen J, Svedung I. Proactive risk management in a dynamic society. Karlstad: Swedish Rescue Services Agency 2000.

12. Cedergren A, Petersen K. Prerequisites for learning from accident investigations--a cross-country comparison of national accident investigation boards. *Saf Sci* 2011;**49**(8):1238-45.

For peer review only

13. Cresswell JW. Qualitative inquiry and research design: Choosing among five approaches. Lincoln: Sage Publications 2007.

14. Stake RE. The art of case study research. Thousand Oaks: Sage Publications 1995.

15. Hollnagel E. Investigation as an impediment to learning. In: Hollnagel E, Nemeth CP, Dekker SWA, eds. Resilience Engineering Perspectives: Remaining Sensitive to the Possibility of Failure, vol. 1. Aldershot: Ashgate Publishing Limited 2008: 259-268.

16. Dekker SWA, Cilliers P, Hofmeyr JH. The complexity of failure: Implications of complexity theory for safety investigations. Safety Science 2011.

17. Cook RI, Render M, Woods DD. Gaps in the continuity of care and progress on patient safety. *BMJ* 2000;**320**(7237):791-4.

18. Flin RH, O'Connor P, Crichton M. Safety at the sharp end: Training non-technical skills. Ashgate Publishing; 2008.

19. Perry SJ, Wears RL, Patterson ES. High-hanging fruit: Improving transitions in health care. Advances in Patient Safety: New Directions and Alternative Approaches 2008;**3**.

For peer review only

1

TITLE PAGE

1. Title of the article:

Mind the gap between recommendation and implementation – principles and learnings in the aftermath of incident investigations. A semi-quantitative and qualitative study in search for factors that lead to successful implementation of recommendations.

2. Full name, postal address, e-mail, telephone and fax numbers of the corresponding author:

Jonas Wrigstad
Department of Anesthesia and Intensive Care
Children's Hospital
Skåne University Hospital Lund
SE-22185 Lund
Sweden
E-mail: jonas.wrigstad@med.lu.se
Phone: +4646706200321
Fax: +4646178219

3. Full names, departments, institutions, city and country of all co-authors:

Johan Bergström, Lund University, Centre for Risk Assessment and Management, Lund, Sweden

Pelle Gustafson, Lund University, Department of Clinical Sciences, Lund, Sweden

4. Keywords:

Management
Patient safety
Adverse events
Investigation

For peer review only

2

ABSTRACT

Objectives: Using incident investigations as a tool for improvement in patient safety management is both a well-established routine, and a responsibility stated by Swedish law. The main intention with the method is to promote certain recommendations that through implementation in the organisation lead to improvement. This study seeks to identify the mechanisms behind successful implementation of such recommendations.

Setting: A university hospital in southern Sweden

Participants: From the hospitals incident reporting system a sample of 55 incident investigations from 2008 to 2010 were selected by staff at the chief medical officer of the organisation. These investigations were initiated by 23 different commissioning bodies and contained 289 separate recommendations. We used a three-stage method: first a content analysis to code the recommendations, then semi-structured interviews with the commissioning bodies focusing on which recommendations that had been implemented and why, and third, a data analysis of the coded recommendations together with data from the interviews.

Results: We found that a clear majority (70 %) of the recommendations presented to the commissioning bodies were targeted towards the micro-level of the organisation. In nearly half (45 %) of all the recommendations, actions had been taken and a clear majority (73 %) of these were at the micro-level. A change at the management position of the commissioning body meant that almost no further actions were taken. Separate actions, free-standing from and independent of the incident investigations, were often taken within the organisation.

Conclusions: We conclude that two principles (”close-in-space” and ”close-in-time”) seem to be important factors when bridging the gap between recommendation and implementation. The micro-level focus is expected because of the method of investigation used. Adverse events trigger organisational interaction independently of incident investigations.

For peer review only

3

Strengths and limitations of this study

- **The results presented in this study shows the strength of using a design that combines content analysis with interviews to thereby get a deeper understanding of the different aspects of the data.**
- **The semi-structured nature of the interviews seemed to make the respondents willing to elaborate and reflect freely on both questions and follow-up questions which resulted in a substantial amount of qualitative data.**
- **The coding scheme in the Content analysis and the categories used in the Data analysis could possibly give a limited perspective of a more complex reality**

INTRODUCTION

When adverse events (AEs) occur in complex socio-technical healthcare systems, it is a challenge – if at all possible - to interpret the underlying causal factors. The importance of using past events to establish organisational learning is a non-controversial claim, but harder to institutionalise in practice.[1] Nevertheless, incident investigations have for decades been routine and seen upon as important tools in safety management, primarily to prevent similar events by promoting recommendations for ensuring continuous improvement.

Different organisations use different methods for their incident investigation activities and a majority, including healthcare, have adopted an underlying accident model in which recommendations made presupposes a stable causal structure of the system.[2, 3] Such a stable causal structure implies that the recommendations are derived by identifying the root cause with no need for relating the specific recommendations to the damaged system as a whole.[2] Johnson argues that the understanding of how certain recommendations are formulated is generally weak.[4] It has been shown that the time spent by the investigators on the activity of providing recommendations is surprisingly short, in comparison to other parts of the process.[5] Furthermore, which factors that govern a successful implementation of recommendations has so far received limited focus in literature.[6, 7]

For peer review only - http://bmjopen.bmj.com/site/about/guidelines.xhtml

The aim of this study was to start filling this knowledge gap by analysing the mechanisms behind successful implementation of recommendations formulated in investigations of incidents in Swedish healthcare. The approach follows Hollnagel's advice to search the positive aspects of safety rather than the negative ones.[8]

BACKGROUND

The Swedish healthcare systems regulatory authority at the time of the study, the National Board of Health and Welfare (NBoHaW), has issued regulations controlling the responsibilities of the different healthcare givers, e.g. using an incident reporting system and performing incident investigations. Swedish law states that the responsibility for patient safety improvement is placed within the organisation itself of the separate healthcare givers.[9, 10] The law also states that if an AE has resulted, or could have resulted, in a severe incident, this should be reported to the regulatory authority for a separate investigation. This investigation, the so called Lex Maria (LM)-investigation, is independent from the incident investigation by the healthcare provider. Deciding whether to report or not to the NBoHaW, is almost exclusively a decision by the chief medical officer (CMO) of the organisation, although the CMO has neither formal legal authority nor responsibility as regards the safety level of the organisation.

A commissioning body (CB) initiates and frames the incident investigation, and is ultimately responsible for the follow-up of the recommendations from the report.
The analysis team, formed by the CB, will consist of at least one healthcare professional, trained in investigating AEs in the Swedish healthcare system. Since 2005, the methodological support for conducting the investigations is distributed by the Swedish Association of Local Authorities and Regions (SALAR), and supported by the NBoHaW.[5] In Swedish healthcare, completed incident investigation reports are, after de-identification, publicly available, as are LM-investigations made by the NBoHaW.

METHODS

We used a three-stage method. First, we did a content analysis to code the recommendations in a sample of 55 incident investigations of AEs in a Swedish university

For peer review only - http://bmjopen.bmj.com/site/about/guidelines.xhtml

5

hospital. We then did semi-structured interviews of CBs focusing on which recommendations that had been implemented and why. Finally, we conducted a data analysis using the coded recommendations together with the interview data, which was used to isolate specific mechanisms contributing to successful implementation of recommendations. Due to the semi-quantitative nature of the study, we did no statistical analyses further than presented.

Content analysis

The first step was to sample a limited amount of completed incident investigations. In collaboration with the CMO at a Swedish university hospital, a frame was set to collect registered AEs, which led to incident investigations, through the incident reporting system of the hospital. The CMO was asked to judge from which year, following 2005 when the introduction of the manual for methodological support from the SALAR was introduced, the hospital had a sufficient and qualified staff of incident investigators who were working within the organisation and familiar with the methodology. Second, at least one year should have passed after completion of the incident investigation to allow a reasonable chance for implementation of recommendations. Third, the selection of investigations should be linked to incidents in which the department of anaesthesia and intensive care at the hospital to some extent was involved. The reason behind this third selection was the main author being an anaesthesiologist, ensuring 1) a comprehensive data set through contacts with important actors, as well as 2) being able to fully understand the cases and investigations, regardless of complexity. This framing resulted in 55 separate incident investigations from January 2008 to December 2010, initiated by 23 different CBs. We also identified the position from where the incident investigation was initiated, as this was the same position where the recommendations would be presented upon completion. Thus, continuity in management was of interest, not individuals.

Using the incident reporting system of the hospital, containing all registered AEs, the completed incident investigations were coupled to potentially existing additional investigations, e.g. LM-investigations.

For peer review only

6

All incident investigation reports and recommendations were numbered as they were received from the office of the CMO. Data from the reports was coded in a scheme focusing on the different CBs at the time of investigation, the ward from which the analysis was commissioned, time spent by the investigation team on conducting the investigation, the number of team members, the number of suggested recommendations, and whether or not the findings of the investigation were reported by the hospital to the NBoHaW (as a LM-investigation).

Rasmussen and Svedung [11] shift the focus to not only include 'what' causal factors that get constructed in the aftermath of adverse events, to also include 'where' in the organisational hierarchy that the constructed causal factors get located. We therefore coded the reports regarding the hierarchical level being the target of recommendation according to a micro-meso-macro perspective.[12] This was done in order to find potential correlations between the hierarchical level and the likelihood of the recommendation being implemented. Micro was judged as a recommendation where it was possible for the CB to implement this entirely within its own department without major constraints, e.g. local procedures, technical skills or staff issues. Meso was judged as a recommendation were the CB had to collaborate with a stakeholder outside of the own department, but within the boundaries of the hospital, e.g. another department or the hospital management. Macro was judged as a recommendation were the boundaries of the hospital had to be crossed, e.g. authorities, politicians, or pharmaceutical companies.

From the written reports it was not possible to gain knowledge to what extent the different recommendations had been implemented or not. This data was added to the coding scheme following the interviews.

Semi-structured interviews

The second part of this study consisted of an interview study with the different CBs at the hospital, to gain a deeper insight into which recommendations had been implemented and why. The interviews were semi-structured, as they focused on specific reports, but with the possibility for the respondents to reflect freely on the questions asked.

For peer review only

7

All of the CBs received written information prior to the interview about the background and aims of the project, as well as the main questions that formed the basis for the interview. All respondents were de-identified and given a random number. Twenty-two of 23 CBs (or their successors) approved through written consent to being interviewed. This made it possible to ask questions of interest in 50 of 55 incident investigation reports, with a total of 254 coded recommendations. Four of the 22 CBs delegated the interview to either the assistant director (2/4), or to the head advisor in patient safety (2/4). The interviews were all carried out during the period of April to September 2012 by the first author (JW) at a place suggested by the respondent. Twenty of the interviews were audio recorded. In 2 of the interviews the respondents did not agree to audio recording and instead extensive notes were taken. All quotations presented here have been translated from Swedish to English by the first author and the quotations are all tagged with the number of the coded respondent.

All interviews included a minimum of three questions (see below). Subsequent questions were asked depending on the answers given by the respondents.

1. Have you taken part of this incident investigation report with attention to the recommendations *before* this study?
2. Which recommendations from the incident report have been *implemented* in the organisation?
3. Have, to your knowledge, any *alternative* actions been taken within the organisation because of the incident investigation report that were not presented as recommendations?

Due to the coincidental fact, that during the time-period framed by the CMO, the hospital had a system were in one part of the hospital, the CB almost exclusively was the CMO, whereas in the other part the CB was the clinical head of the department, the interviews had to be conducted somewhat differently. A CMO in a Swedish hospital cannot also be clinical head of a department at the same time, or vice versa. Therefore, in the investigation reports where the CB was the CMO, interviews were also done with clinical heads of departments involved. The reason for this was to get a deeper knowledge of how far the implementation process had developed regarding recommendations to the different departments.

For peer review only

8

Data analysis

The analysis was conducted as a generalization using the interview data to seek naturalistically generalized factors explaining the results of the content analysis.[13, 14]

Before the naturalistic generalization commenced, the coding scheme was extended with the different answers regarding if action had been taken or not on specific recommendations. We used three categories in this study:

A – actions have been taken and initiated/completed regarding the recommendation

B – actions have not been taken regarding the recommendation

C – no knowledge if actions have been taken

Due to the fact that many clinical heads of department would be interviewed about the same specific incident investigation, a decision had to be made if answers would fall into categories that were contradictory. It then became important to follow up with asking the clinical head answering according to category A how and when the particular recommendation had resulted in actions.

We analysed interview data in particular search for generalized patterns: Why did the distribution between micro, meso and macro look the way it did? What was the connection with successful implementation, and why? What aspects of successful implementation of recommendations were not captured in the content analysis, and why? Did, or did not, factors such as the position of the CB or the time spent by the analysis team, influence the likelihood of the suggested recommendations being implemented?

RESULTS

Content analysis of incident investigation reports

39 of the 55 AEs were subject to both an incident investigation by the hospital and to a LM-investigation by the authorities, suggesting that the degree of severity in a majority of the events had surpassed a limit stated by the authorities. Implementations of recommendations from the LM-investigations were not analysed in this study.

For peer review only

9

The CBs of the 55 incident investigations were similarly distributed between the CMOs (n=29) and the heads of departments (n=26).

The average number of team members per investigation was 2.7, and the duration of an investigation varied from 12 man-hours to 150 man-hours, where both of these findings are in the same range that have been shown by Rollenhagen for typical investigations in patient safety.[5]

Among the 55 incident investigations 289 separate recommendations were identified, where 5 recommendations were not coded because of our uncertainty of what the investigators actually meant in their written report. Thus 284 coded recommendations remained and, as mentioned previously, questions of interest were raised during the interviews in 254 of these coded recommendations where their distribution in the organisational hierarchy is shown in table 1.

Table 1. The distribution of the recommendations according to the three hierarchical levels as well as whether they have been reported as having resulted in actions.

Hierarchical level	No of recommendations for which actions have been taken	No of recommendations for which actions have not been taken	No of recommendations for which there is no knowledge if actions have been taken	Total number
Macro	1	6	0	7
Meso	30	26	16	72
Micro	82	53	40	175
Total number	113	85	56	

In the following sections both semi-quantitative and qualitative data, including the categories from the content analysis and quotations from the interviews of the CBs, will be presented in order to identify mechanisms important (or not) for the successful implementation of recommendations.

For peer review only

10

Management continuity

From the interviews, we found that the hospital, since commissioning the investigations, had appointed a new CMO for all of the 29 incident investigations, and new clinical heads of department in 41 cases of the broadened interview material, where one incident investigation could involve a number of interviews with directors of department. When the question "*have you taken part of this incident investigation report with attention to the recommendations before this study?"* was raised, the new CMO had taken part of 3/29 and the new clinical heads of department 6/41. As one of the CBs noted:

> "One could have a system where the CMO is a bit more meticulous and does a follow-up of the incident investigations to see what happened. It could be more of supervising position than it is today, but there is no time for that. That would probably be a part time job in itself or a substantially increased workload."(11.)

Overall, the respondents were concerned about the lack of knowledge regarding incident investigation reports completed before taking on their management position.

> "I have not informed myself about past events, but that illustrates two important things, according to myself, that we use the results from the incident investigations too scantly and there is not enough follow-up. (…) But I think the most important matter is – these are historical cases and if one hasn't been clinically involved it's a problem with commitment – that there is a follow-up on the recommendations so that something does happen…" (2.)

Nowhere in the organisation, we found a good system of keeping record of which actions had been taken on the recommendations from the incident investigations. To a varying degree, the respondents had been able to find information on what kind of actions had been taken. As shown in table one, after all interviews with the interpretation approach mentioned above, we found that for 45 % of the recommendations actions had been taken, for 33 % actions had not been taken, and for 22 % of the recommendations made our respondents were not able to tell us whether actions had been taken.

For peer review only

11

> ”No, note that there isn’t a single one of these incident investigations I’ve known about. (…) I’ve talked to my assistant director, dr (…), and to the member of staff responsible for the departments incident reporting, nurse (…), to collect some information” (22.)

The position of the CB

Whether the CB was a CMO or head of department did not seem to influence the process of implementation. When it was a CMO actions had been taken in 55/128 (43 %) of completed investigations, and when it was a head of department it was 58/126 (46 %).

> “…and when many departments are involved in the adverse event it doesn’t work with just one director of department being the commissioning body…But it’s also complicated to hand this over to the chief medical officer because it often has a tendency to come to nothing when many actors are involved. Who takes the responsibility?” (11.)

Micro, meso or macro

As seen in table one, in the cases where actions have been taken (A), the interviews showed that a majority were on a micro-level.

> ”Yes, actions have been taken. We’ve written a new document about this procedure, that I have right in front of me, so that I can remember everything that has been done…and regarding *that* matter we’ve put it on the checklist and the surgeon must ask before surgery whether procedures have been followed.(…) This was a very easy and straightforward thing to solve, one could say. There was one thing that had gone wrong and we tried to fix it …and others weren’t involved”. (4.)

The event itself as a trigger for change

In 19 of 50 cases, the interviews showed that the AE had initiated organisational actions that were not presented as recommendations in the reports. It seemed that the incident investigations in these cases worked more as an incentive to change things, but on the initiative from the management, rather than from the analysis team.

> ”So you see, despite numerous meetings and brain storming back and forth I still believe that all of this was completely off target. (…) So in this case we did this formalistic play,

For peer review only

12

which was good, but then we resigned a bit. Thereafter, among the senior colleagues, we drew a pragmatic conclusion and went on. There was someone who quoted Shakespeare at the time; 'Much ado about nothing' or something like that…" (10.)

"…then some of us decided, within the department, to start a minor recurring training course. (…) You see, it often comes down to quite strange results if we aren't part of the changing process. (…) And when the colleagues 'over there' gained some knowledge about this matter, things definitely got better, at least from my point of view. (…) Today this way of working is almost self-driven and I see it as a result completely independent of the investigation." (16.)

The time spent by the investigation team

In 7 of 50 incident investigations it was not possible to find out the time spent by the investigating team on conducting the investigation. In 43 of 50 investigations, which consisted of 217 recommendations, there was a spread in duration from 12 man-hours to 150 man-hours. By arbitrarily grouping different investigations after time spent on the investigation, we wanted to examine if duration was a factor for implementation and at what level.

The groups consisted of short duration <40h, medium duration 41 to 80h and long duration >81h. We found that in the group with short duration, actions had been taken in 25/55 with 21/25 actions being on a Micro-level, in the group with medium duration actions had been taken in 43/116 with 28/43 actions on Micro-level, and in the long-duration group actions had been taken in 24/46 with 20/24 actions on Micro-level. Thus, with the methods in use in the hospital, the duration of an investigation differed with more than a factor 10, without time consumption seeming to influence the actions that were taken to meet the recommendations made, or at what level.

DISCUSSION

This study has several strengths and limitations. The results presented shows the strength of using a design that combines content analysis with interviews to thereby get a deeper understanding of the different aspects of the data. The semi-structured nature of the

For peer review only

interviews seemed to make the respondents willing to elaborate and reflect freely on both questions and follow-up questions which resulted in a substantial amount of qualitative data. The coding scheme in the content analysis and the categories used in the data analysis could possibly give a limited perspective of a more complex reality. It could be argued that an investigation is not completed before a formal post-implementation follow-up has been done. However, we have not studied the effect of the implemented recommendations, since the gap between recommendation and implementation was the focus.

We dare not have an opinion as regards the possibility to draw general conclusions from this study. However, we have no reason to expect that our findings are unique to the speciality studied (anesthesiology), or the type of hospital studied (university hospital), and thus believe that our findings could be valid for other Swedish hospitals, and possibly also hospitals in countries with a similar system for investigations of AEs.

This study shows that a clear majority of the recommendations presented to the CB are targeted towards the micro-level of the organisation, even when the investigating team spent a considerable amount of time. Our conclusion of why this is the case is not that the micro-level is necessarily the most meaningful target of intervention, but rather that it reflects the investigating teams' understanding of how incidents happen. This is well summarised in Hollnagel's two principles: WYLFIWYF ("What You Look For Is What You Find") and WYFIWYF ("What You Find Is What You Fix").[2, 7, 15] In this study what the investigators have been looking for, in terms of causal relations, is intimately linked to the linear causation constructed not as much by the investigators themselves as by the method available to them. A linear incident model, as the one inherent in the method provided by the SALAR [5], used in the investigations studied, identifies certain problems as relevant targets of intervention. This is not the first study supporting the conclusion that linear incident investigation methods tend to locate causal explanations at the micro-level of the organisational hierarchy [16] although we also see that what is found is not always fixed and it is not always the recommendations written in the reports that decide what will be fixed.

In the literature regarding healthcare system safety, much focus has been directed towards the sharp end, such as transition in care: the changing of shifts, change of ward and change

For peer review only - http://bmjopen.bmj.com/site/about/guidelines.xhtml

of level in care.[17-19] Based on the findings from this study, we argue that in order to understand successful implementation of recommendations after analyses of AEs, important factors are also to be found at the blunt end of the organisation, such as changes in the positions in management or management continuity. Thereby saying that if the individual in a management position was a successor to the original commissioning body, there was almost no previous knowledge of an existing completed investigation, and understandably, hardly any further actions were taken than had already been done by the predecessor.

Consequently, two principles - “close-in-space” and “close-in-time” - seem to be important factors when closing the gap between recommendation and implementation using a model such as in this university hospital.

The finding that the event itself triggers organisational interventions regardless of the recommendations made in the incident investigation requires some further elaboration. Not only could this be interpreted as a finding that lowers the organisational mandate of the analysis process, but it also complicates the process of conducting the analysis, especially if using a model assuming a stable causal structure. If organisational interventions get initiated simply as a result of the event, this means that the organisation essentially goes through a qualitative process of change as a result of the event. Consequently this implies that the organisation is qualitatively different after the event than it was before.

Since the organisation did not keep record of which recommendations had been taken action on, our findings rely almost entirely on the responses from the interviews. This in itself could give a hint of some uncertainty about the reliability of the analysis, but could on the other hand raise some concern about how incident investigations are used in the organisations work with patient safety improvement. Based on the interviews, nearly half of all the recommendations had been implemented, almost regardless of the organisation’s perceived severity of the adverse event. A clear majority of these recommendations were at the micro-level with hardly any difference in actions taken depending on the management position of the CB.

For peer review only

15

The focus on success mechanisms also becomes a focus on system vulnerabilities and potential improvement. The finding that suggested actions are most likely to get implemented following a “close in time” and “close in space”-principle can indeed guide future work to improve the system of learning from AEs in Swedish healthcare. We suggest that future research and projects, aimed at improving the quality of the system, focus on 1) developing means to institutionalise an organisational memory of AEs and the analyses following them so that the system becomes less sensitive to management continuity, 2) pushing the target of analysis following AEs towards levelling the balance of suggested actions more between the micro, meso and macro levels of the organisation. This requires a method for analysis which directs the focus of analysis to interactions and relations at higher organisational levels, but also investigation teams with basic competence in safety science and interpretation of complex systems. Furthermore, 3) the gap between investigation team and investigated organisation. Based on the finding that actions beyond those suggested in the formal analyses are taken following AEs, we suggest future work to be done studying how the dialogue between analysis team and the organisation being the target of the analysis can be enhanced, and 4) learning from incidents beyond the formalised system. We suggest future research to be conducted with a focus on possible storytelling of past incidents in healthcare organisations. Is it actually so that there are many lessons learned that never get brought up in formal investigations, but that nevertheless becomes a part of the organisational memory and everyday behaviour?

CONCLUSIONS

This study seeks to interpret factors that lead to successful implementation of recommendations suggested in incident investigations following AEs in a Swedish university hospital. Based on the findings, we conclude that continuity in management is an important factor for successful implementation of recommendations (= close-in-time), as is that a clear majority of the recommendations presented in the studied incident investigations are targeted towards a micro-level of the organisation where the same applies to the recommendations that actually get implemented (= close-in-space). The micro-level focus of the investigations is expected given the linear causal model underlying the method of analysis. For recommendations to be targeted towards meso- and macro-levels of the organisation the model used for investigation needs to seek explanatory factors at the level

For peer review only - http://bmjopen.bmj.com/site/about/guidelines.xhtml

of organisational interactions and relations. Furthermore, the AE itself triggers organisational interventions regardless of the recommendations made in the incident investigations, and the time spent by the investigation team or the position of the CB were both factors which did not stand out as contributing to successful implementation of recommendations.

FOOTNOTES

Acknowledgments: The authors would like to thank the staff at the office of the CMO for their contribution by providing all the necessary incident investigation reports.

Funding: The project was partially funded by a grant, but with no further involvement, from The Swedish National Patient Insurance.

Formatted: Font: Not Bold

Contributors: JW developed the study, collected, analysed and interpreted the data and wrote the manuscript. JB and PG contributed in the study design, analysis of the data and revision of the manuscript.

~~**Acknowledgments:** The authors would like to thank the staff at the office of the CMO for their contribution by providing all the necessary incident investigation reports.~~

Competing interests: None.

Ethics approval: The study was approved by The Regional Ethical Review Board in Lund, Sweden.

~~**Funding**: The project was partially funded by a grant, but with no further involvement, from The Swedish National Patient Insurance.~~

Data sharing: No additional data available.

For peer review only

17

REFERENCES

1. Woods DD, Cook RI. Nine steps to move forward from error. *Cogn Technol Work* 2002;**4**(2):137-44.

2. Lundberg J, Rollenhagen C, Hollnagel E. What-You-Look-For-Is-What-You-Find--The consequences of underlying accident models in eight accident investigation manuals. *Saf Sci* 2009;**47**(10):1297-311.

3. Rasmussen J, Nixon P, Warner F. Human error and the problem of causality in analysis of accidents [and discussion]. *Philos Trans R Soc London B Biol Sci* 1990;**327**(1241):449-62.

4. Johnson CW. Failure in safety-critical systems: a handbook of accident and incident reporting. University of Glasgow Press, Glasgow, Scotland, 2003:541-623

5. Rollenhagen C, Westerlund J, Lundberg J et al. The context and habits of accident investigation practices: A study of 108 swedish investigators. *Saf Sci* 2010;**48**(7):859-67.

6. Cedergren A. Implementing recommendations from accident investigations: A case study of inter-organisational challenges. *Accid Anal Prev* 2013, Apr;**53**:133-41.

7. Lundberg J, Rollenhagen C, Hollnagel E. What you find is not always what you fix—how other aspects than causes of accidents decide recommendations for remedial actions. *Accid Anal Prev* 2010;**42**(6):2132-9.

8. Hollnagel E. Resilience engineering in practice: A guidebook. Ashgate Publishing, Ltd.; 2011:xxix-xxxix

9. The Swedish Patient Safety Act (SFS 2010:659). http://www.riksdagen.se/sv/Dokument-Lagar/Lagar/Svenskforfattningssamling/Patientsakerhetslag-2010659_sfs-2010-659/ (accessed 4 Nov 2013)

10. The National Board of Health and Welfares management system for systematic quality work (SOSFS 2011:9). http://www.socialstyrelsen.se/sosfs/2011-9/allaversioner2011-9 (accessed 4 Nov 2013)

For peer review only

11. Rasmussen J, Svedung I. Proactive risk management in a dynamic society. Karlstad: Swedish Rescue Services Agency 2000.

12. Cedergren A, Petersen K. Prerequisites for learning from accident investigations--a cross-country comparison of national accident investigation boards. *Saf Sci* 2011;**49**(8):1238-45.

13. Cresswell JW. Qualitative inquiry and research design: Choosing among five approaches. Lincoln: Sage Publications 2007.

14. Stake RE. The art of case study research. Thousand Oaks: Sage Publications 1995.

15. Hollnagel E. Investigation as an impediment to learning. In: Hollnagel E, Nemeth CP, Dekker SWA, eds. Resilience Engineering Perspectives: Remaining Sensitive to the Possibility of Failure, vol. 1. Aldershot: Ashgate Publishing Limited 2008: 259-268.

16. Dekker SWA, Cilliers P, Hofmeyr JH. The complexity of failure: Implications of complexity theory for safety investigations. Safety Science 2011.

17. Cook RI, Render M, Woods DD. Gaps in the continuity of care and progress on patient safety. *BMJ* 2000;**320**(7237):791-4.

18. Flin RH, O'Connor P, Crichton M. Safety at the sharp end: Training non-technical skills. Ashgate Publishing; 2008.

19. Perry SJ, Wears RL, Patterson ES. High-hanging fruit: Improving transitions in health care. Advances in Patient Safety: New Directions and Alternative Approaches 2008;**3**.

For peer review only
